# Physiologic and metabolic characterization of a new marine isolate (BM39) of *Pantoea* sp. producing high levels of exopolysaccharide

**DOI:** 10.1186/1475-2859-12-10

**Published:** 2013-01-29

**Authors:** Silvia Silvi, Paolo Barghini, Arianna Aquilanti, Belen Juarez-Jimenez, Massimiliano Fenice

**Affiliations:** 1 Dipartimento di Scienze Ecologiche e Biologiche and Laboratorio di Microbiologia Marina Applicata, CONISMA (Consorzio Interuniversitario Scienze del Mare), University of Tuscia, Viterbo, 01100, Italy; 2 Departamento de Microbiologia, Facultad de Farmacia, Campus de Cartuja, University of Granada, Granada, 18071, Spain; 3 Laboratorio di Microbiologia Marina Applicata, CONISMA (Consorzio Interuniversitario Scienze del Mare), University of Tuscia, Viterbo, 01100, Italy

**Keywords:** *Pantoea* sp., Halophilic bacterium, Flow cytometry, Biolog, Exopolysaccharide production

## Abstract

**Background:**

Marine environments are the widest fonts of biodiversity representing a resource of both unexploited or unknown microorganisms and new substances having potential applications. Among microbial products, exopolysaccharides (EPS) have many physiological functions and practical applications. Since EPS production by many bacteria is too scarce for practical use and only few species are known for their high levels of production, the search of new high EPS producers is of paramount importance. Many marine bacteria, that produce EPS to cope with strong environmental stress, could be potentially exploited at the industrial level.

**Results:**

A novel bacterium, strain BM39, previously isolated from sediments collected in the Tyrrhenian Sea, was selected for its production of very high levels of EPS. BM39 was affiliated to *Pantoea* sp. (Enterobacteriaceae) by 16S rRNA gene sequencing and biochemical tests. According to the phylogenetic tree, this strain, being quite far from the closest known *Pantoea* species (96% identity with *P. agglomerans* and *P. ananatis*) could belong to a new species. EPS production was fast (maximum of ca. 21 g/L in 24 h on glucose medium) and mainly obtained during the exponential growth. Preliminary characterization, carried out by thin layer and gel filtration chromatography, showed that the EPS, being a glucose homopolymer with MW of ca. 830 kDa, appeared to be different from those of other bacteria of same genus*.* The bacterium showed a typical slightly halophilic behavior growing optimally at NaCl 40 ‰ (growing range 0-100 ‰). Flow cytometry studies indicated that good cell survival was maintained for 24 h at 120 ‰. Survival decreased dramatically with the increase of salinity being only 1 h at 280 ‰. The biochemical characterization, carried out with the Biolog system, showed that MB39 had a rather limited metabolic capacity. Its ability, rather lower than that of *P. agglomerans*, was almost only confined to the metabolization of simple sugars and their derivatives. Few alcohols, organic acids and nitrogen compounds were partially used too.

**Conclusions:**

Strain BM39, probably belonging to a new species, due to its remarkable EPS production, comparable to those of known industrial bacterial producers, could be suggested as a new microorganism for industrial applications.

## Background

Oceans and seas are the widest sources of biological and chemical diversity representing a prolific reserve of unexploited and/or unknown microorganisms [1,2]. Thus, marine environments are great resources of new substances having potential applications in pharmaceutical, feed and food, fine chemicals and enzyme industries [2,3]. The search of new microorganisms, having unique physiological and metabolic capabilities, aids to better comprehend the ecosystem and provides opportunities to discover new compounds of commercial importance. This is particularly true for marine bacteria that have been less studied than their terrestrial counterpart and are often underrated or completely ignored by many scientists [4,5].

Among the microbial products, exopolysaccharides (EPS) have many important physiological functions and various practical applications deductible from their roles in nature.

These high molecular weight polymers represent essential components of the secreted extracellular material and are involved in various cell function such as: cell protection from freezing, dehydration and antimicrobial agents [6-9]; adhesion to surfaces, other organisms and biofilm production [10]; support in pathogeny and virulence [11,12]; inhibition of biofilm formation [13,14]; storage of reserve carbon sources [10].

EPS find applications in environmental biotechnology being employed in soil and water bioremediation, decontamination and detoxification [15-18]. Moreover, they are used in pharmaceutical/biomedical [19,20], cosmetic [21], chemical [22,23] and food industries [24,25].

The amount of EPS produced by many bacteria, few grams per liter, is too low for their practical use. By contrast, only few species are known for their high levels of production. Among them, strains of *Xanthomonas campestris*, *Bacillus polymyxa*, *Klebsiella pneumonie* and *Sfingomonas elodea* are the most studied and only few are used at the industrial level [16,26-29].

Different microorganisms produce EPS with diverse composition and having different characteristics leading to their employment in diversified ambits [12,16]. In addition, same microorganism could release EPS with different composition when grown in different conditions [17]. In this context, the search of new high EPS producers is still important to find new applications or better fit traditional uses. Moreover, strain physiologic and metabolic characterization is extremely useful to understand and optimize microbial productions [30,31].

In marine environment many bacteria, producing EPS to cope with strong environmental stress and to survive adverse conditions [32-34], represent promising sources of species to be exploited at the industrial level.

In this study, we report on the detailed metabolic characterization of a new slight halophilic marine bacterium producing high levels of exopolysaccharide. The strain was identified as *Pantoea *sp. by 16S rRNA gene sequencing and biochemical tests. Time course of EPS production and partial chemical characterization of the polymer are also reported. In addition, physiologic adaptation to salinity is also studied by both cultural methods and flow cytometry.

## Results and discussion

### Strain identification

The isolate, subjected to 16S rDNA sequence analysis (1266 bp), was affiliated to the genus *Pantoea.* Its sequence, GeneBank accession number “BankIt1581807 Pantoea KC163803”, matched with entries with similarities ranging from 96 to 98%. However, matching with known species of *Pantoea* was 96% only; thus, due to the low similarity, BM39 assignment to the species level was not possible.

**Figure 1:**
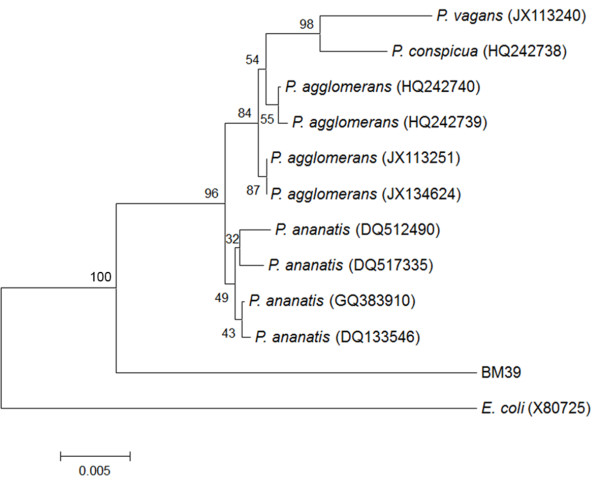
**Phylogenetic tree of *Pantoea *species based on 16S rDNA sequences.** The tree, based on 14 sequences and 1300 positions, has been generated using neighbor-joining algorithm and maximum composite likelihood model and calculated using Mega4 program. Bootstrap values from 1000 pseudo-replicates are shown.

In addition, considering the broad phylogenetic distance from the most similar *Pantoea* species, the strain could belong to a new species. Figure 1 reports the phylogenetic relationships, based on alignments with the most similar sequences of 16S *Pantoea* species, as obtained by comparison with Blastn analysis. Due to evident inaccurate species attribution, some sequences have not been included in the dendrogram; the outgroup constituted by *E. coli* was added according to literature [35-37]. The phylogenetic analysis showed that BM39 constituted an external cluster quite far from the most similar species, *P. ananatis* and *P. agglomerans,* organized in two separate groups. Within the *P. agglomerans* group there was a further cluster of *P. conspicua*, and *P. vagans* (Figure 1)*.*

The uncertain affiliation of BM39 was observed by the Biolog system too. The information obtained did not consent the attribution to species included in the database being *P. agglomerans,* the closest species with 51% of similarity only.

### Metabolic characterization

Preliminary tests showed that strain BM39, as generally reported for *Pantoea*[35,36,38,39] is a mobile, gram negative, catalase positive and oxidase negative rod (0.42±0.15 – 2.87±1.0 μm).

### Growth and physiological state at different salinities

Traditionally, strict definition of “marine microorganism” implies that a marine species must be found only in marine environments [40,41]. Even if many species are just confined in marine environments, others, widely diffused in terrestrial environments, present strains that are well adapted to marine conditions [30]. Thus, it is difficult to understand if a microorganism, isolated from sea samples could be defined as “marine”. Actually, the isolate could be a strict marine microorganism, an adapted strain from other environments or a microorganism accidentally found still alive in the sea but non-adapted to marine conditions.

Sea salinity in BM39 sampling area is around 38 ‰ all the year [42,43] and was measured at 37.8 ‰ during sampling.

**Figure 2:**
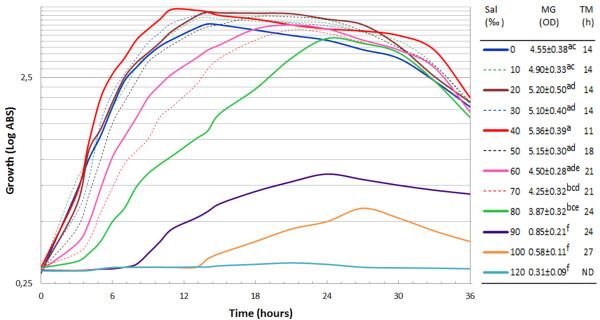
**Time course of growth of *Pantoea *sp. BM39 cultivated for 36 h on LB containing different concentration, 0-120 ‰ step 10 ‰, of NaCl measured spectrofotometrically (OD_600_).** Table legend reports OD_600_ and the time of maximal growth at the various concentrations of NaCl. Data followed by same superscript letter are not significantly different (P<0.05) by the Tukey test. Legend table reports: Sal = Salinity; MG = maximum growth and TM = time to reach maximum growth. Values in same column followed by at least one identical superscript letters are not significantly different by the Tukey test (P<0.01).

*Pantoea* sp. BM39, tested at different salinities ranging from 0 to 120 ‰, grew optimally at NaCl 40 ‰ stating at least its adaptation to marine environment. However, no statistical differences were recorded for maximal growth in the range 0-60 ‰. By contrast, differences were significant in relation to the time necessary to reach maximal growth (Figure 2). Starting from 70 ‰, significant differences were recorded for maximal growth also. BM39 grew up to 100 ‰ but above 80 ‰ growth was very limited and strongly delayed (Figure 2).

The microorganism, thus defined as slight halophilic, appears well adapted to a rather broad range of salinity but growth far from optimal conditions required more time probably for more complex homeostasis regulation.

More detailed information concerning homeostasis and physiological state of each bacterial cell, submitted to different conditions of salinity, had been obtained by flow cytometry in the range 0-280 ‰.

**Figure 3:**
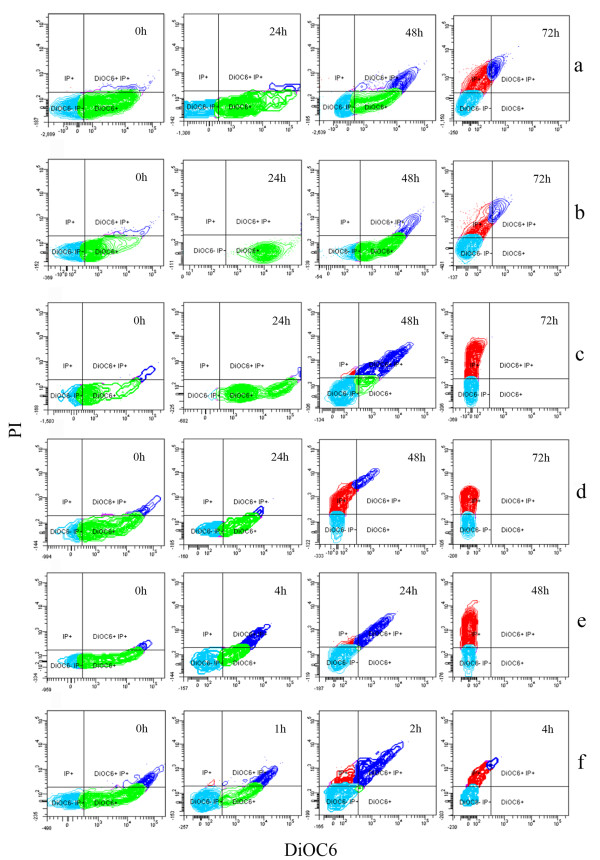
**Flow cytometry of BM39 grown for 72 h on LB containing different concentrations of NaCl, 0 ‰ (a), 40 ‰ (b) 80 ‰ (c), 120 ‰ (d), 200 ‰ (e) and 280 ‰ (f), and stained with DiOC6 and PI.** Only more significant samples are shown. Green spots = DiOC6 positive cells showing high membrane polarization; Light blue spots= DiOC6 and PI negative showing cells in latency; Dark blue spots = DiOC6 positive and PI positive showing cells starting to loose membrane polarization and to acquire PI; Red spots = PI positive showing dead cells.

Figure 3 reports the physiological state of BM39 cells, at different salinities and incubation times, in terms of membrane polarization and ratio between live and dead cells as determined by the differential staining with DiOC6 and PI, respectively. At 0 h, the bacterium physiological state is quite similar for all the tested NaCl concentrations (Figure 3a-f). Some cells, with low membrane polarization, could be considered still in a latent state (scarce DiOC6 and no PI), while the majority, showing well polarized membranes, presented active and stable physiological conditions (strong DiOC6). Only few dying cells were recorded particularly in samples at higher salinity (scarce PI). It is expected that cells, grown in favorable conditions of nutrients and chemico-physical parameters, pass from latency to the active state starting their metabolic activities. This situation, evidenced by staining with DiOC6 only, persists until favorable conditions are maintained. If favorable conditions are not established or in case of nutrients depletion, viable cells pass to the latent state, loosing membrane polarization, before starting to die. Such cells lose DiOC6 and start to assume PI while dead cells are strongly PI stained only. All these physiological conditions and the transition among the various situations were well evidenced for BM39 in Figure 3.

In this context, remarkable differences were recorded, during the experiment progression, in relation to salinity. As expected, optimal conditions were confirmed at 40 ‰. In fact, this is the sole situation showing all cells in complete viable state (strong DiOC6, only) after 24 h of incubation. Cells started to die, for possible initial starvation, around the 48 h to be in advanced dead phase at 72 h (Figure 3b).

Similar behavior was recorded both at 0 and 80 ‰ even if signs of cell sufferance were more evident at 48 h, in particular at 80 ‰ (Figure 3a, c). The progressive increase of salinity proportionally determined the increase of cell sufferance. This is particularly evident at 280 ‰; in this case, after only 2 h, almost all cells were died or dying (Figure 3f).

**Figure 4:**
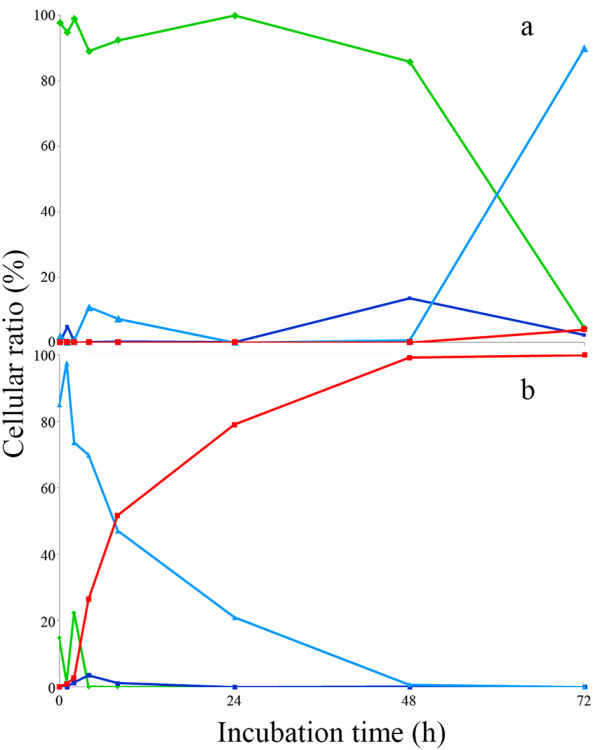
**Time course of cell populations fractions of *Pantoea *sp. BM39, grown for 72 h on LB containing 40 ‰ (a) and 280 ‰ (b) of NaCl and stained with FDA and PI, as revealed by flow cytometry.** Green line = FDA positive cells showing high viability; Light blue line = FDA and PI negative showing cells in latency; Dark blue line = FDA positive and PI positive showing cells starting to loose viability and to acquire PI; Red line = PI positive showing dead cells.

Same situation was recorded using a different combination of fluorescent dyes (FDA+PI). Figure 4 reports the time course of the various fractions of BM39 cell populations showing different physiological states (latency, active viability, dying and dead) in two opposite conditions of salinity, 40 (optimal) and 280 ‰ (worst). At 40 ‰, almost all the cell, after a short period of latency, showed high viability till nutrients were available (48 h); starvation started thereafter (Figure 4a). By contrast, at 280 ‰ intense cell sufferance was recorded already after 1 h and cells started to exponentially die thereafter (Figure 4b).

### Metabolism of different carbon sources

**Figure 5:**
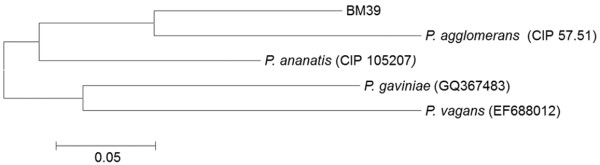
**Dendrogram of metabolic similarities among *Pantoea *sp. BM39 and other *Pantoea *species generated using neighbor-joining algorithm and calculated using Mega4 program.** Similarity has been calculated based on 50 different carbon sources.

The metabolic abilities of BM39, in relation to the use of 95 carbon sources, were tested by the Biolog system. The strain showed a rather limited metabolic competence being able to use only 24 compounds (Table 1). Among them, the majority were simple sugars or derivatives. Some organic and amino acids and few other nitrogen compounds were metabolized too. Even with diversified competence, similar low metabolic capacity was recorded for *P. vagans*[35], while *P. agglomerans* showed wider aptitude (Biolog database). A limited metabolic competence indicates a rather specialized strain with low eco-versatility as reported for other microorganisms [3,30,44]. Comparison, between BM39 and other *Pantoea* species, in relation to the metabolic abilities, is not easy due to the scarce information available and to the different methodologies used. However, we compared the use of 50 carbon sources with data obtained in literature [35,37,45]. Figure 5 reports a dendrogram showing the metabolic relationships between BM39 and other *Pantoea* species. Our strain, that appeared equidistant from *P. agglomerans* and *P. ananatis* under the phylogenetic point of view (Figure 1), was found much more similar to *P. agglomerans* at the metabolic level being in the same cluster. This could be explained by the great metabolic diversity within the genus *Pantoea*[36,45].

**Table 1:** **Comparison between the metabolic competences of *Pantoea *sp. BM39 and other *Pantoea *species as revealed by the Biolog system**

**Carbon source**	**BM39**	***Pa***	***Pv***
α-Cyclodextrin, dextrin, glycogen, N-Acetyl-D-galactosamine, adonitol, i-erythritol, L-fucose, lactulose, D-raffinose, D-sorbitol, xylitol	**-**	**-**	**-**
N-acetyl-D-glucosamine, L-arabinose, D-fructose, D-galactose, α-D-glucose, maltose, D-mannitol, D-mannose, sucrose, D-trehalose,	**+**	**+**	**+**
D-arabitol, D-psicose, turanose	**-**	**+**	**-**
D-cellobiose, gentiobiose	**-**	**-**	**+**
m-inositol	**+**	**-**	**+**
α-D-lactose, D-melibiose, β-A-26-methyl-D-glucoside	**+**	**+**	**-**
L-rhamnose	**-**	**+**	**+**
Succinic ac. methyl-ester, acetic ac., formic ac., D-galactonic ac. Lactone, D-glucosaminic ac., α-OH-butyric ac., β-OH-butyric ac., γ-OH-butyric ac., p-OH-phenylacetic ac., itaconic ac., α-keto butyric ac., α-keto glutaric ac., α-keto valeric ac., propionic ac., quinic ac., D-saccharic ac., sebacic ac., bromosuccinic ac., succinamic ac., glucuronamide	**-**	**-**	**-**
Pyruvic ac. methyl ester, D-gluconic ac., D, L-lactic ac.	**+**	**+**	**-**
Cis-aconitic ac., D-glucuronic ac., D-galacturonic ac.	**-**	**+**	**-**
Citric ac., succinic ac.	**-**	**+**	**+**
Malonic ac.	**-**	**-**	**+**
L-alaninamide, L-alanylglycine, L-asparagine, glycyl-L-aspartic ac., glycyl-L-glutamic ac., L-histidine, OH-L-proline, L-leucine, L-ornithine, L-phenylalanine, L-pyroglutamic ac., L-threonine, D,L-carnitine, γ-amino butyric ac., urocanic ac.,	**-**	**-**	**-**
L-glutamic Ac.	**+**	**+**	**+**
D-alanine, L-alanine, L-aspartic ac., L-proline, D-serine	**-**	**-**	**+**
L-serine	**-**	**+**	**-**
Phenyethylamine, putrescine, 2-aminoethanol, 2,3-butanediol	**-**	**-**	**-**
Glycerol	**+**	**+**	**+**
Tween 40	**-**	**-**	**+**
Tween 80	**-**	**-**	**+**
Inosine, uridine, thymidine	**+**	**+**	**-**
D,L-α-glycerol phosphate	**-**	**+**	**-**
α-D-glucose-1-phosphate, D-glucose-6-phosphate	**+**	**+**	**-**

Legend: *Pa* = *P. agglomerans* (Biolog database)*; Pv* = *P. vagans* (Brady et al., [2009]).

### Production of EPS and partial polymer characterization

**Figure 6:**
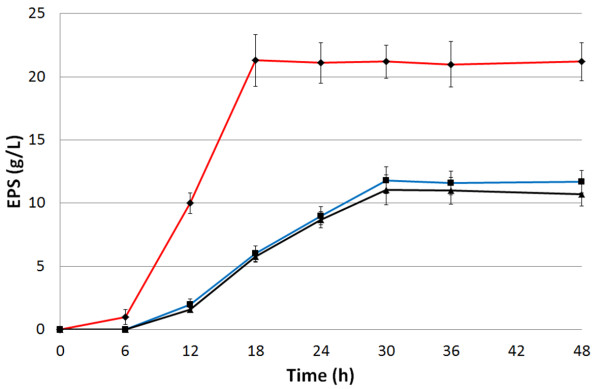
**Time course of EPS production by *Pantoea *sp. BM39 grown on EMF (black line), EMG (red line) and EMS (blue line) for 48 h in shaken cultures at 28°C and 180 rpm.**

Growth and EPS production by BM39 was tested using rather common carbon sources (sucrose, glucose and fructose) at a quite high concentration to induce high production (Figure 6) [8,26,28]. As for the bacterial biomass, there was no statistical difference among the various media. Maximal EPS production (21.30±2.03 g/L) was obtained on glucose (EMG) after 18 h of incubation. On both sucrose and fructose EPS release was definitely lower and delayed, being 11.82±1.06 and 11.05±1.17 g/L at 30 h, respectively. All other kinetic parameters, such as yield and productivity, were highest on EMG (Table 2). The superior yield recorded in EMG means that in this medium the bacterium was able to better convert the substrate into EPS (Y_P/S_) and the biomass was more efficient (Y_P/X_). In other words, a lower amount of biomass contributed to higher EPS production. The highest productivity in EMG is particularly interesting in view of possible application at the industrial scale.

**Table 2:** **Kinetic parameters of EPS production by *Pantoea *sp. BM39 cultivated in shaken cultures on different media**

	**X (g/L)**	**T (h)**	**P (g/L)**	**Y**_**P/S**_	**Y**_**P/X**_	**R (g/Lh)**
EMF	11.94±1.02^a^	30	11.05±1.17^a^	0.14	0.93	0.37±0.04^a^
EMG	13.72±1.42^a^	24	21.30±2.03^b^	0.27	1.55	0.89±0.09^b^
EMS	13.06±0.94^a^	30	11.82±1.06^a^	0.15	0.91	0.39±0.04^a^

Legend: X = maximum biomass; T = time of maximum EPS production; P = EPS production; Y_P/S_ = yield (product/substrate); Y_P/X_ = yield (product/biomass); R= productivity at time of maximum EPS production. Data are the means of three independent experiments ± SD. Values in same column followed by the same superscript letters are not significantly different (P<0.01) by the Tukey test.

Since medium has not been optimized yet and process had been carried out in shaken flasks, the EPS production by BM39 could be considered already very high. It is worth noting that, as reported for many other processes, microbial productions could be strongly improved by accurate medium formulation and culture condition optimization [31,46-49]. Our strain production could be considered quite good also in relation to other already studied bacteria. Various known producers release just few g/L of EPS and rarely exceed the amount of 10 g/L [26,27,29,50-52]. Few others, produce much higher levels of EPS comparable with those of BM39 or even higher. However, the high production was often obtained after optimization to increase strain performance [28,53]. For example, *P. agglomerans* (*Enterobacter agglomerans)* strain CRDA 312 produced 27.5 g/L of EPS [53] but the production was achieved in stirred bioreactors that generally consent better performances in relation to same process carried out in shaken flasks [31].

Preliminary EPS characterization, carried out by thin layer chromatography after acid hydrolysis, showed that the polymer was constituted only by monomeric units of glucose (data not shown). This justifies the better performance of EMG in comparison with the other media tested. Very likely, glucose is directly used to assemble the biopolymer while fructose and sucrose need a bioconversion before EPS formation. Apparent molecular weight of the polymer, determined by gel permeation chromatography, was 830 kDa.

It is worth noting, that other known species of *Pantoea* produce EPS with different composition and characteristics. For example, *P. stewartii* is note to produce “stewartan” a heteropolymer of glucose and galactose [54]. *P. agglomerans* KFS-9 produces a heteropolymer constituted by arabinose, glucose galactose and gulcuronic acid with a molecular weight of 760 kDa [55]. However, BM39 production was obtained on media containing glucose, fructose or sucrose. It is possible that, on other carbon sources, EPS with different composition and characteristics could be obtained.

## Conclusions

*Pantoea* sp. strain BM39, probably belonging to a new slightly halophilic marine species, showed rather broad euryhaline behavior growing up to ca. 100 ‰ of salinity. The bacterium was able to rapidly produce quite high levels of a homopolymeric glucose EPS that, being different from those of other bacteria, could have different applications. EPS production is comparable with those of known industrial strains and, taking into account that the process has not been optimized yet, BM39 could be considered very promising for the exploitation at the industrial level.

## Methods

### Chemicals

Plate Count Agar (PCA), Yeast extract (YE); Bacto-Tryptone (BT), Mycological Peptone, Luria Bertani broth (LB) and LB agar (LBA) were from Difco (USA). All other chemicals were of analytical grade.

### Microorganism and culture conditions

*Pantoea* sp. BM39 was previously isolated from sediments sampled at 20 m deep in the Tyrrhenian Sea off the coast of Civitavecchia, Roma, Italy [3]. During the study the strain was maintained on PCA at 4°C and subcultured when necessary.

Inocula were prepared suspending some loopful of the bacterium from a PCA plate in 250 ml Erlenmeyer flasks containing 50 ml of LB. Flasks were shaken cultured overnight at 180 rpm and 28°C.

Media for EPS production were as follows (g/L): NaNO_3_, 5.0; KCl, 0.5; KH_2_PO_4_, 1.0; FeSO_4_ x 7H_2_O, 0.01; CaCO_3_, 35.0; Mycological Peptone, 1.0 added with glucose 80.0 (EMG) or sucrose 80.0 (EMS) or fructose 80.0 (EMF).

For EPS production, 250 ml Erlenmeyer flasks, filled with 50 ml of each medium, were added with the bacterial inoculum produced as above (0.150 OD_600_) and shaken cultured (180 rpm, 28°C) for 72 h. Samples were collected every 6 h. Experiments were done in triplicate.

Media for determination of optimal salinity for growth were prepared adding the necessary amounts of NaCl (from 0 ‰ to 120 ‰, step 10 ‰) to BT 1% and YE 0.5% (LB without NaCl).

For determination of optimal salinity for growth, 250 ml Erlenmeyer flasks, filled with 50 ml of each medium, were added with the bacterial inoculum produced as above (0.300 OD_600_) and shaken cultured (180 rpm, 28°C) for 36 h. Samples were collected every 1 h during the first 15 h and every 3 h thereafter. Experiments were done in triplicate.

All media were autoclaved at 121°C for 20 min.

### Morphological, physiological and biochemical characterization

Tests were carried out on early exponential phase cells from cultures grown at 28°C. Morphological characterization was done using Gram stained cells. Gram staining was carried out using a commercial kit (Merck, Germany) following manufacturer’s instructions. Strain dimensions were obtained using a Leitz Laborlux 11 microscope bearing a micrometric ocular calibrated with a micrometric slide (Leitz Wetzlar, Germany). Catalase and oxidase tests were performed as previously described [56,57]. Briefly: for oxidase activity, Kovacs reactive (1% of N,N,N,N tetrametil-p-phenylenediamine in water) was added to a fresh colony. After 60 seconds, develop of violet color means positive reaction. For catalase, H_2_O_2_ (3%) was added to a fresh colony: bubbles of O_2_ production meant a positive reaction.

Extended metabolic competences were investigated testing the strain ability to use 95 different compounds (including carbohydrates, carboxylic acids, polymers/oligomers, amines/amides, aminoacids and other compounds) as sole carbon source by the “Biolog” system [30,58,59] according to the manufacturer’s directions; results were interpreted with the most recent Biolog Microlog database (Biolog, Hayward, CA, USA).

### Strain identification and phylogeny

The strain was identified by analysis of the sequences of the gene encoding for the 16S rRNA. Bacterial genomic DNA was extracted and used for amplification by polymerase chain reaction. Products of amplification were sequenced and compared with databases sequences. Taxonomical information was also obtained by the above mentioned Biolog data base.

### DNA extraction and polymerase chain reaction for amplification of the 16S rRNA gene

BM39 grown for 24 h on PCA plates, was used for genomic DNA extraction by thermal shock as follows [60]: a single colony suspension (in 14 μl of sterile deionized water) was heated at 100°C for 5 min, immediately cooled in ice and centrifuged at 4000 *g* for 3 min. The supernatant was used for PCR reaction. Amplifications were performed in a reaction mixture (final volume 25 μl) containing 2x BioMix (BioLine GmbH, Germany), 15–20 ng/μl of DNA template and 5 pmol/μl of the following universal primers 1389r (ACGGGCGGTGTGTACAAG) and 63f (CAGGCCTAACACATGCAAGTC) (Sigma-Aldrich, USA). Amplification was carried out using a MiniCycler™ (MJ Research, USA) equipped with a heated lid as follows: denaturation at 95°C for 5 min; denaturation at 95°C for 45 s; annealing at 55°C for 1 min; extension at 72°C for 90 s; final extension at 72°C for 7 min; cold-storage 4°C. Step 2, 3 and 4 were repeated for 30 cycles.

PCR products were visualized by electrophoresis on agarose gel (1.0%) prepared with 0.50 g of agarose (Starlab GmbH, Denmark) dissolved in 50 ml of TAE buffer 1X (40 mM Tris-acetate, 1 mM EDTA, pH 8.3, Brinkmann Instruments, Inc., USA) added with 5 μl of GelRed (10,000x, Biotium, USA). Loading was carried out by adding 1 μl of Loading Dye (6x, New England Biolabs, USA) to 5 μl of each sample. The DNA Ladder GeneRuler™ 100 bp (FERMENTAS, Lithuania) was used to quantify PCR products dimension by comparison. The products were purified using Nucleospin Extract kit (Macherey-Nagel, Germany). Sequencing reactions were performed by Macrogen sequencing service (Macrogen Inc., Korea). Sequence assembly was done using the software Chromas (version 1.5 2009, Technelysium Pty Ltd, Australia). Sequences with high similarity available in NCBI GenBank were identified using BLASTn search.

BM39 sequence was deposited to NCBI/GenBank database with the “BankIt1581807 Pantoea KC163803” accession number.

### Alignment and tree reconstruction

Automatic alignment was first carried out using CLUSTALX [61], then exported to MEGA4 [62] and improved manually. Phylogenetic tree was reconstructed by neighbor-joining algorithm and maximum composite likelihood model. The robustness of the phylogenetic inference was estimated using the bootstrap method [63] with 1000 pseudo-replicates.

### Exopolysaccharide determination and partial characterization

Bacterial cells and CaCO_3_ were removed by centrifugation (15 min at 6000 rpm). After removing possible residual CaCO_3_ from culture supernatant with 1 N HCl, EPS concentration was determined by precipitation at 4°C adding 2 volumes of absolute ethanol. Precipitated EPS was filtered on pre-weighed Whatman GF/D discs, filters were then dried at 95°C for 24 h, cooled into a desiccator and weighed.

For characterization, EPS was recovered by precipitation as above. Precipitate was collected and re-dissolved in distilled water: the procedure (precipitation, centrifugation and re-dissolution in water) was repeated twice. The final aqueous solution was dialyzed against distilled water (24 h at 4°C) freeze-dried, and weighed [8].

EPS was hydrolyzed with 2 N sulfuric acid at 100°C for 3 h. Then, the solution was neutralized with 1 N NaOH and filtered (Whatman discs, 0.45 μm). Sugar components were identified by thin-layer chromatography (TLC): sugar standards were used for identification.

Thin-layer chromatography (TLC) was performed on silica gel plates 60 F254 (Merck, Darmstadt, Germany) saturated with 0.5 M KH_2_PO_4_ using a solvent system of lactic acid (7.4 g/L of distilled water), 2-propanol and acetone in a ratio of 5:1:10. Sugar spots were visualized by spraying the plates with a solution made up of 96 ml of 0.2% naphthoresorcinol solution in ethanol plus 4 ml of concentrated sulfuric acid and incubation at 100°C for 5 min.

EPS apparent molecular weight was determined by gel permeation chromatography as described previously with slight modifications [64]. Briefly, a chromatographic Superose-6 column connected to a FPLC system (Pharmacia) was used for determination after calibration with commercial dextrans (48.6, 80.9, 147.6, 273.0, 409.8, 667.8 and 1400.0 kDa by Sigma-Aldrich).

### Flow cytometry analysis

The study has been carried out by the “Servicio de Biologia Fundamental, Centro de Instrumentacion Cientifica”, University of Granada, Granada, Spain using a FACSCanto II cytometer (Becton Dickinson, San José, CA, USA). The cytofluorimeter (CF) was equipped with three laser sets (405, 488, and 625 nm) and detectors for forward-scatter, side-scatter and eight fluorescence colours. Acquisition from the CF and data analysis was done by the FACSDiva v6.1.3 software (Becton Dickinson).

Cells grown for 24 h at 28°C on LBA plates, containing 40 ‰ of NaCl, were harvested and suspended (10^8^ cell/ml) in 40 ‰ NaCl in distilled water. From this concentrated suspension the necessary amount of cells were taken and re-suspended in LB, containing different amount of NaCl (range 0-280 ‰, step 40 ‰), to reach a final bacterial concentration of 10^6^ cell/ml. The various suspensions were incubated in an orbital shaker (28°C and 180 rpm) for 72 h. Samples, taken after 0, 1, 2, 4, 8, 12, 24, 48 and 72 h, were added with propidium iodide (PI) and 3,3-dihexylocarbocyanine iodide (DiOC6) or fluorescein diacetate (FDA) to reach final concentration of 1.0, 0.005, 2.0 μg/ml, respectively. After 15 min of incubation at 28°C stained samples were submitted to the multi-parameter FC and analysed.

The physiological state of the bacterium individual cells was characterized adding different combinations of the fluorogenic dyes as follows. Presence of both an intact polarized cytoplasmic membrane and active transport systems, essential for a fully functional cell, was tested by the addition of PI and DiOC6. PI binds to DNA, but cannot cross an intact cytoplasmic membrane, and DiOC6 accumulates intracellularly when membranes are polarized or hyperpolarized [30,65,66]. In addition, cell viability has been tested using the combination of PI and FDA [67]. FDA is actively transported into the viable cells and is converted by membrane esterases into a fluorogenic compound (emission at 530 nm): cell having good homeostasis (viability) are fluorescent. As said PI enters damaged membranes and indicate dying or dead cells.

### Statistical analysis of data

One-way analysis of variance (ANOVA) and pair-wise multiple comparisons procedure (Tukey test) were carried out using the software SigmaStat (Jandel Scientific, CA, USA).

## Competing interests

The authors declare that they have no competing interest.

## Authors’ contribution

MF and PB carried out the design of this study. MF overviewed fermentations and data analysis. SS was responsible of fermentations, performed determinations and EPS characterization. MF and PB performed data analysis. AA performed the phylogenetic study and determinations. BJ performed the study at the cytofluorimeter. All authors participated in writing and critical manuscript review. All authors have read and approved the manuscript.
